# Language and Sensory Neural Plasticity in the Superior Temporal Cortex of the Deaf

**DOI:** 10.1155/2018/9456891

**Published:** 2018-05-02

**Authors:** Mochun Que, Xinjian Jiang, Chunyang Yi, Peng Gui, Yuwei Jiang, Yong-Di Zhou, Liping Wang

**Affiliations:** ^1^Key Laboratory of Brain Functional Genomics, Institute of Cognitive Neuroscience, School of Psychology and Cognitive Science, East China Normal University, Shanghai 200062, China; ^2^Department of Physiology and Neurobiology, Medical College of Soochow University, Suzhou 215123, China; ^3^Institute of Medical Psychology, Medical College of Soochow University, Suzhou 215123, China; ^4^Institute of Neuroscience, Key Laboratory of Primate Neurobiology, CAS Center for Excellence in Brain Science and Intelligence Technology, Chinese Academy of Sciences, Shanghai 200031, China; ^5^Department of Neurosurgery, Johns Hopkins University, Baltimore, MD 21218, USA; ^6^NYU-ECNU Institute of Brain and Cognitive Science, NYU Shanghai and Collaborative Innovation Center for Brain Science, Shanghai 200062, China

## Abstract

Visual stimuli are known to activate the auditory cortex of deaf people, presenting evidence of cross-modal plasticity. However, the mechanisms underlying such plasticity are poorly understood. In this functional MRI study, we presented two types of visual stimuli, language stimuli (words, sign language, and lip-reading) and a general stimulus (checkerboard) to investigate neural reorganization in the superior temporal cortex (STC) of deaf subjects and hearing controls. We found that only in the deaf subjects, all visual stimuli activated the STC. The cross-modal activation induced by the checkerboard was mainly due to a sensory component via a feed-forward pathway from the thalamus and primary visual cortex, positively correlated with duration of deafness, indicating a consequence of pure sensory deprivation. In contrast, the STC activity evoked by language stimuli was functionally connected to both the visual cortex and the frontotemporal areas, which were highly correlated with the learning of sign language, suggesting a strong language component via a possible feedback modulation. While the sensory component exhibited specificity to features of a visual stimulus (e.g., selective to the form of words, bodies, or faces) and the language (semantic) component appeared to recruit a common frontotemporal neural network, the two components converged to the STC and caused plasticity with different multivoxel activity patterns. In summary, the present study showed plausible neural pathways for auditory reorganization and correlations of activations of the reorganized cortical areas with developmental factors and provided unique evidence towards the understanding of neural circuits involved in cross-modal plasticity.

## 1. Introduction

Cortical structures that are deprived of their normal sensory input may become responsive to the stimulation of adjacent receptors, a process that is generally known as cross-modal plasticity or cross-modal reorganization [1]. In human brain imaging studies, there is growing evidence showing that, in early bilaterally deaf adults, the superior temporal cortex (STC) may experience cross-modal recruitment of different visual inputs, such as visual motion [2–8], biological motion [9–11], sign language [11–19], and silent speech reading [15, 20–23]. Animal models have also confirmed the dystrophic change that occurs when the auditory cortex fails to develop typically due to the absence of auditory input [24–28].

Visual-related responses in the STC of deaf subjects could result from long-term auditory deprivation (e.g., missing auditory sensory input) but could also be caused by other dynamic cognitive functions (e.g., sign language learning) [1, 12, 16, 19, 29, 30]. In the previous studies, STC activity was found to positively correlate with the duration of deafness or the age at cochlear implantation [2, 18, 31–35], suggesting that functional reorganization was likely to take place in the auditory cortex over a considerable period of time. A functional magnetic resonance imaging (fMRI) study showed that STC activation was highly correlated with speech reading fluency, but not with the duration of sensory deprivation [36], indicating that functional compensation of sensory deprivation did not require slow progressive colonization of the STC by visual inputs, but instead rapidly modulated by the preexisting latent connectivity from high-level language-related cortical areas. Thus, for the reorganization of STC, potentially both bottom-up signals (e.g., from the visual cortex) and top-down modulation (e.g., from the associative frontal-temporal areas) could contribute to such cross-modal activity [30]. Meanwhile, a magnetoencephalography study showed that the left frontotemporal network, including the STG, was activated during lexicosemantic processing in the congenitally deaf individuals, but not responsive to the early sensory visual processing, suggesting a more top-down modulation from high-level language-related regions [37].

Although it is clearly known that the STC responds to various visual stimuli in deaf people, the neural mechanisms underlying this cross-modal plasticity are still not fully understood. There are questions remaining to be answered. First, how do developmental factors (e.g., the duration of deafness or the learning of sign languages) in deaf people constrain or promote the reorganized activity in the auditory cortex? Second, how do the bottom-up and top-down two neural pathways contribute to cross-modal activation? Third, does the STC integrate inputs from different pathways, or does it keep them functionally segregated?

In the present study, using fMRI, we aimed to directly compare cross-modal activity and whole-brain functional connectivity in subjects when they were viewing a general stimulus (checkerboard) representing the bottom-up input from the visual cortex and language-related stimuli (words, sign language, and lip-reading) denoting the both bottom-up from visual regions and top-down signals from associative cortical areas. Nineteen profoundly deaf (congenital) subjects, 15 residual hearing subjects with a hearing aid, and 15 hearing subjects were recruited to investigate how behavioral factors (e.g., the duration of hearing loss and age at sign language learning) affected cross-modal activity. This study also aimed to investigate possible sources of cross-modal activation by applying dynamic causal modeling (DCM) [38] and representational similarity analysis (RSA) [39]. We hypothesized that the reorganized STC activity by a checkerboard was mainly induced through a feed-forward network and that activity provoked by language-related stimuli was instigated from both feed-forward and feedback components, but relied more on the feedback regulation. Furthermore, it was considered that the STC activities responsive to the two pathways were likely to be functionally segregated.

## 2. Materials and Methods

### 2.1. Participants

Thirty-four early-deaf subjects (14 males; mean age: 20.8 years old) and 15 hearing controls (7 males; mean age: 20.3 years old) participated in the study. The deaf participants were from the Shanghai Youth Technical School for the Deaf (http://www.shlqj.net/longxiao), and their information on the history of hearing loss, hearing aid use, and sign language use was documented through an individual interview (Table 1). All participants were healthy, had a normal or corrected-to-normal vision, were not taking psychoactive medications, did not have a history of neurological or psychiatric illness, took classes at the high-school level, and had normal cognitive functions. In the residual hearing group, most participants communicated by a combination of two or three strategies, which included spoken language (13 out of 15), lip-reading (8 out of 15), and sign language (11 out of 15), while most of the profound deaf (15 out of 19) communicated only via sign language. The ethical committee at East China Normal University in China approved the experimental procedure. All participants gave their informed and written consent according to the Declaration of Helsinki and were paid for their participation. The 15 hearing subjects were recruited from East China Normal University in China and had no learning experience of sign language or lip reading. The groups were matched for age, gender, handedness, and education.

Suitable deaf participants were selected by means of hearing threshold pretests, conducted within the 2 weeks preceding the fMRI experiment. To facilitate a preliminary screening of the subjects, deaf participants self-reported their level of hearing loss on the basis of their audiologists' diagnoses. Hearing thresholds of all the participants were then measured at the Institute of Speech and Hearing Science, Shanghai. Thresholds were assessed monaurally for both ears, either with or without a hearing aid, at 250, 500, 1000, 2000, 4000, and 8000 Hz, using steps of 5 dB. According to the International Hearing Impairment Classification Standard [40], we divided the 34 deaf participants into two groups in terms of their hearing loss level: profoundly deaf (>90 dB, *n* = 19; in average, the left hearing is 106.8 ± 2.5 dB; the right hearing is 106.7 ± 2.4 dB) and residual hearing (<75 dB, *n* = 15; the left hearing is 73.6 ± 5.5 dB; the right hearing is 76.1 ± 4.4 dB) (Table 1).

### 2.2. Visual Stimuli

**Figure 1:**
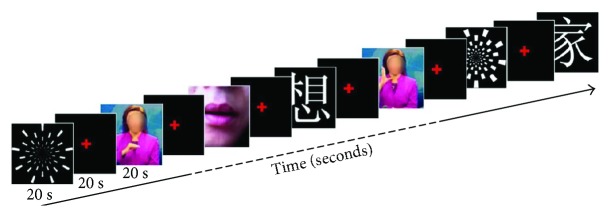
An example session of experimental paradigm. Subjects were presented alternating blocks of four different visual stimuli: checkerboard, words, sign language, and lip-reading. The order of presentation was pseudorandomly assigned. For each subject, there were 20 blocks (four stimuli × five repetitions), with each stimulus presented for 20 s and the black screen sustained for 20 s as an interval. The whole experiment was separated into three sessions for the purpose of avoiding subject fatigue. Throughout a block, subjects were asked to either fixate on a red cross at the center of the screen or concentrate on the visual stimuli. Questions were asked at the end of the experiment to ensure that subjects had paid attention to the visual stimuli (Supporting information).

Four different visual materials were presented to participants, a checkerboard pattern to act as a general visual stimulus, and three visual stimuli with language content: words, sign language, and lip-reading (Figure 1, which also see details in Supporting information (available here)). All stimuli were pseudorandomly presented using a block design (Figure 1). Within each block, only one type of stimulus was presented. Each block lasted 20 s and was followed by a 20 s interblock interval. During the 20 s visual presentation, the stimuli were played at a similar rate. During the 20 s interval, a red cross with a black background was presented at the center of the screen and participants were asked to maintain their gaze on the cross. Per subject, twenty blocks in total were included. That is, each type of stimulus was repeated for five times. The blocks were separated into three sessions (6 or 7 blocks per session), with a 5 min intersession interval for rest.

Checkerboard stimuli were presented at 1280 × 1024 pixels. Each image was presented for 1 s. Word stimuli were composed of 80 Chinese characters (monosyllable) chosen from the List of Frequently Used Characters in Modern Chinese written by the State Language Commission of China. Each character was written in white on a black background and presented as a stimulus for 1 s using a font and size of SimSun 36. For sign language stimuli, five sentences were chosen and expressed by a female presenter moving her hands and arms without facial expression. The video was presented at a resolution of 1024 × 768 pixels, and each sentence lasting 10 seconds was repeated twice within the same block (20 seconds). The presenter's face in the video was masked to avoid potential interference from the lip-reading. For lip-reading stimuli, consecutive frames of a feminine face pronouncing disyllable Chinese words were presented at a moderate speed. The disyllable words were chosen from the Lexicon of Common Words in Contemporary Chinese by the Commercial Press. Both sign language and lip-reading stimuli were displayed at a rate similar to that used for the word and checkerboard stimuli (~1 Hz). The questionnaire data after scanning showed that all the participants were able to view the stimuli clearly and understand the content of each stimulus (Supporting information).

### 2.3. Experiment Procedure

The fMRI experiment was approved by the Shanghai Key Laboratory of Magnetic Resonance at East China Normal University. Before scanning, the experimental paradigm and scanning procedures were introduced to the deaf participants through a professional signer. They were asked to stay focused on stimuli and were told that they would be asked questions later after the scan to ensure that attention had been paid to the stimuli. Visual stimuli were displayed on a half-transparent screen hung around 285 cm away from the participant's eyes and displayed via a LCD projector (Epson ELP-7200L, Tokyo, Japan). The participant viewed the screen through a mirror. The participant's right hand was placed on a button box connected to a computer so that the participant was able to press a button as a sign that he/she wished to withdraw at any stage of the experiment or scan, without having to give a reason.

After scanning, all participants were asked to complete a feedback questionnaire about the content of the experiment and their subjective experiences, to ensure that they were paying attention during the experimental sessions. They were also asked to give ratings on a 3-point scale (3  =  all stimuli were very clear, 1  =  all stimuli were not clear) to ensure both the clarity of visual stimuli presented and their full engagement in the experiment. Additionally, participants had to describe what they had just seen between trials, the frequency of checkerboard flashing, and the meaning of simple words, sign language, and lip-reading sentences used during the experiment. We did not intend to control the complexity of the language stimuli. The rating scores from stimulus categories did not significantly differ from each other (one-way ANOVA, *p* > 0.3).

### 2.4. Data Acquisition

The fMRI was performed on a 3-T TimTrio (Siemens, Erlangen, Germany) scanner. During scanning, the participant's head was immobilized using a tight but comfortable foam padding. To avoid nonspecific activation, the participant was asked not to make any sort of response or read aloud during the scan. When presented with visual stimuli, the participant was required to concentrate on the presentation but was not required to perform any mental task or physical operation. Ear defenders were used for all residual and hearing participants throughout the whole procedure. Each participant underwent a T1-weighted structural MR scan (3-D FLASH), with 1 mm-thick slices, a repetition time (TR) of 1900 ms, an echo time (TE) of 3.42 ms, a flip angle of 9°, and a field of view (FOV) of 240 × 240 mm. FMRI was performed using echo planar imaging (EPI) sequences with the following parameters: 32 axial slices acquired in an interleaved order; TR, 2000 ms; TE, 30 ms; voxel size, 3.75 × 3.75 × 3.75 mm; flip angle, 70°; and FOV, 240 × 240 mm. A total of 147 sessions (78,400 volumes) were collected from 49 participants.

### 2.5. Preprocessing

The first two volumes of each run were discarded to allow for T1 equilibration effects. Data were analyzed using SPM8 (Wellcome Trust Centre for Neuroimaging, London, UK) running within Matlab 7.10 (Mathworks Inc., Natick, MA, USA). The image data preprocessing followed standard SPM8 preprocessing procedures and included slice timing correction, realignment for the correction of motion artifacts, coregistration to the participant's structural T1 image, normalization to the Montreal Neurological Institute (MNI) template, and smoothing with a Gaussian kernel of [8  8  8] full width at half maximum. No participants were discarded from the analysis. The head movements were less than 3.75 mm.

### 2.6. Cross-Modal Activation Analysis

A first-level analysis approach was adopted for the block-design fMRI data using SPM8. In this step, a general linear model encompassing the design and contrasts at the individual subject level was created. The model contained all the information on different conditions, onsets, and durations for all the scans combined across a subject. The twelve predictors included [1–4] the onsets of the four conditions (checkerboard, words, sign language, and lip-reading) in the profoundly deaf group, [5–8] the onsets of the four conditions in the residual deaf group, and [9–12] the onsets of the four conditions in the hearing group. These twelve events were modeled as delta functions convolved with the canonical hemodynamic response function and its temporal and dispersion derivatives. Head motion parameters derived from realignment were also included in the model as covariates of no interest.

The weighted sum of the parameter estimates from the individual analysis was represented as contrast images that were used for the group analysis using a random effect model. The contrast images obtained from the individual analyses represented the normalized condition-related increment of the MR signal of each subject, with the visual stimulus presentations compared with the resting baseline period (stimuli > baseline). The second-level group analysis of the three participant groups (Group: profoundly deaf, residual hearing, and hearing) in the four experimental conditions (Condition: checkerboard, words, sign language, and lip-reading) was performed using SPM. Each contrast image from the relevant condition was firstly submitted to a one-sample *t*-test at the group level for the whole brain to examine the cross-modal activations in the auditory cortex in individual groups. Then, to identify the differences between groups and conditions, a two-way ANOVA with two main factors: Group and Condition, was conducted for the whole brain using a general linear model. To define the regions of interests (ROIs) for following analyses, the peak voxels were selected within the STC (Brodmann areas 41 and 42) in the right hemisphere of the whole-brain map showing a significant main effect of Group (peak at $\left[\begin{array}{ccc} 66 & -27 & 13 \end{array}\right]$) and within language-related brain regions: the left anterior temporal cortex (ATC, peak at $\left[\begin{array}{ccc} -57 & 7 & -9 \end{array}\right]$) and left inferior frontal gyrus (IFG, peak at $\left[\begin{array}{ccc} -47 & 22 & 13 \end{array}\right]$) in the map showing the Condition effect. A spherical ROI with a 10 mm radius was then generated and centered on the peak voxel. The mean percent signal change for each participant was extracted from the first-level analysis using the Marsbar software tool (http://marsbar.sourceforge.net).

### 2.7. Correlation Analysis

In the residual hearing group, most participants communicated by a combination of two or three strategies, which made the analysis of their language learning experience complicated. In the profoundly deaf group, language experience of four participants was not available. Therefore, only 15 profoundly deaf participants were included in the correlation analysis. For the same reason, only the profoundly deaf group was examined to be compared with the hearing group in the functional connectivity analysis and dynamic casual modeling (descripted below). To test the hypothesis that the sign language experience would modulate cross-modal reorganization, we examined the activity in the right superior temporal cortex (STC; using the ROIs defined in the STC showing the Group effect). Spearman's rank tests for correlations between STC activity and the duration of deafness or between STC activity and the age of learning sign language were performed.

### 2.8. Functional Connectivity Analysis

A functional connectivity analysis was performed to search for brain areas showing significant differences between the profoundly deaf and hearing groups, with the right STC as a seed region (the same ROI in the above analyses). Functional connectivity analyses were performed using CONN-fMRI Functional Connectivity SPM [41]. EPI images that had been preprocessed as described but had undergone no further statistical analysis were used. Connectivity strength was calculated over the visual presentation period. Before the subject-level analysis, standard preprocessing and depositing procedures using the default settings of the CONN toolbox were performed on the EPI data using the BOLD signal derived from white matter masks and cerebrospinal fluid, as well as motion correction parameters from the realignment stage of the spatial preprocessing as covariates of no interest. The data were further band-pass filtered between 0.008 and 0.09 Hz. For each subject, bivariate regression coefficients were estimated to represent the total linear temporal association between the BOLD signal of the ROIs and the rest of the brain. The subsequent analysis compared correlation strengths by a two-sample *t*-test (FDR, *p* < 0.05 corrected) on the beta images from the group analysis to examine the differences between the profoundly deaf and the hearing groups at a whole-brain level. To identify the task specificity in each stimulus condition, a further two-sample *t*-test (FDR, *p* < 0.05 corrected) on the beta images of differences between the groups was performed to examine the differences between the checkerboard condition and the three language conditions.

### 2.9. Dynamic Causal Modeling

Six different models regarding the language-related visual inputs in deaf participants were compared. These models mainly tested whether STC activations were induced by language stimuli receiving the feedback modulation from IFG and ATC and the feed-forward signal from the primary visual cortex (V1) (see Results). Each model was composed of four regions: IFG, ATC, STC, and V1. The extrinsic input (visual stimulation) always entered the system via the V1. The main differences among the models involved the connections among brain regions: specifically, (1) a model with feedback or feed-forward connections between IFG/ATC and STC, (2) a model with both feed-forward connections between V1 and STC and between V1 and ATC, and (3) a model with only feed-forward connections between V1 and STC or between V1 and ATC. The models were split into two classes of families. The first class tested if models with or without feedback (IFG/ATC to STC) were more likely to explain the data. The family with feedback from IFG/ATC to STC included models [1], [3], and [5], and the family without feedback included models [2], [4], and [6]. The second class tested if models fitted the data which explained the connections between V1 and STC, including V1 to STC (models [1] and [2]), V1 to both STC and ATC (models [3] and [4]), or V1 to only ATC (models [5] and [6]). A group analysis (*p* < 0.001, FDR *p* < 0.05 corrected) of deaf participants (profoundly deaf and residual hearing groups) was conducted to investigate the voxels most significantly activated across all three language-related stimuli in areas of left V1, STC, ATC, and IFG. Specifically, the peak intensities of four regions were identified at V1 $\left[\begin{array}{ccc} 0 & -78 & -3 \end{array}\right]$, STC $\left[\begin{array}{ccc} -63 & -48 & 9 \end{array}\right]$, ATC $\left[\begin{array}{ccc} -57 & 7 & -9 \end{array}\right]$, and IFG $\left[\begin{array}{ccc} -48 & 22 & 13 \end{array}\right]$. The principle eigenvariety (time series) was extracted from the volumes of interest that centered at the coordinates of the nearest voxels within a sphere of 8 mm radius (ROI). Based on the estimated model evidence of each model, using SPM8, random effect Bayesian model selection then calculated the “exceedance probability.” When comparing model families, all models within a family were averaged using Bayesian model averaging and the exceedance probabilities were calculated for each model family.

### 2.10. Representational Similarity Analysis (RSA)

The analysis of neural activity within ROIs was conducted with the RSA toolbox (http://www.mrc-cbu.cam.ac.uk/methods-and-resources/toolboxes/) [39]. Both the primary visual area and STC were selected as ROIs defined anatomically by using WFU PickAtlas [42]. We compared the condition-wise patterns amongst fMRI t-maps for the four types of visual stimuli: checkerboard (nonlanguage), words, sign language, and lip-reading (language). Per subject, the representational dissimilarity matrixes (RDMs) comprised correlation distances (1 correlation coefficient) between the images from the blocks for each condition in both the profoundly deaf and the residual hearing group, which yielded a 4 × 4 matrix. The four conditions were separated into two categories: nonlanguage (checkerboard) and language (words, sign language, and lip-reading). We then compared the correlation coefficient in the three pairs between the nonlanguage and the language conditions (category C-L: checkerboard versus words, checkerboard versus sign language, and checkerboard versus lip-reading) with the three pairs within the language conditions (category L-L: words versus sign language, words versus lip-reading, and sign language versus lip-reading) for each subject. In the individual ROIs, the similarities of the two categories were tested statistically (*t*-test, *p* < 0.05) in both the profoundly deaf and the residual hearing groups. As there was no plasticity in the auditory cortex with most of visual stimuli in the hearing group, the RSA analysis did not include such group of participants.

## 3. Results

### 3.1. Brain Activations in Auditory Areas in Response to Visual Stimuli

**Figure 2:**
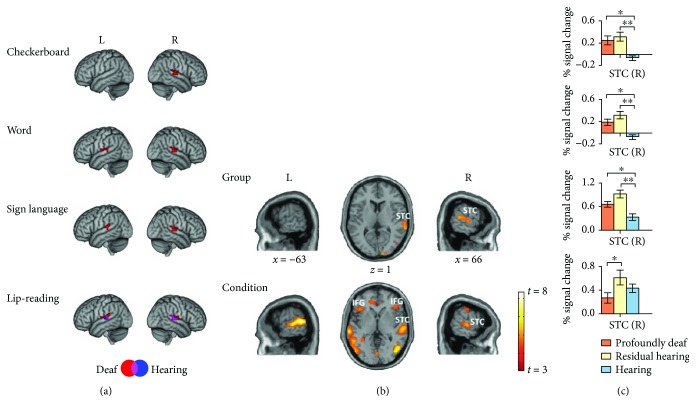
Activations in the superior temporal cortex in response to visual stimuli. (a) Group-level activities for the four visual stimuli are displayed in MNI (Montreal Neurological Institute) standard space. Lateral views of left and right hemispheres showing activity for the checkerboard, word, sign language, and lip-reading stimuli in deaf (red, including both the profoundly deaf and the residual hearing groups, *n* = 34) and hearing (blue, *n* = 15) groups within the brain regions of Brodmann areas (BAs) 41 and 42. The deaf group showed a response to the checkerboard in the right STC region (*p* < 0.001, FDR *p* < 0.05 corrected). Bilateral STC activations were found under the word and sign language conditions in deaf subjects (*p* < 0.001, FDR *p* < 0.05 corrected). Both the deaf and the hearing groups showed bilateral activations for the lip-reading stimuli (*p* < 0.001, FDR *p* < 0.05 corrected). The coordinates and voxel sizes are listed in detail in Table 2. (b) Brain activation to the main effect of Group and Condition are projected on lateral and top views of the brain (*t* > 3.0, *p* < 0.001, FDR *p* < 0.05 corrected). (c) Percent signal change in the regions of interest (10 mm sphere surrounding the peak voxel at $\left[\begin{array}{ccc} 66 & -27 & 13 \end{array}\right]$ in the right STC of the brain map showing the Group effect) of the profoundly deaf (orange, *n* = 19), residual hearing (yellow, *n* = 15), and hearing (blue, *n* = 15) groups. ^∗^ *p* < 0.05 (Student's *t*-test), ^∗∗^ *p* < 0.01. Error bars indicate 1 standard error. L: left hemisphere; R: right hemisphere; STC: superior temporal cortex; IFG: inferior frontal gyrus.

We first examined cross-modal activation in the STC of both the deaf and the hearing groups at the group level for each condition (Table 2). We found that the STC was significantly activated by all of the visual stimuli (*p* < 0.001, cluster level *p*_FDR_ < 0.05 corrected; Figure 2(a)) in the deaf participants. The visual stimuli with language content activated the STC bilaterally, and the checkerboard only induced the STC activation in the right hemisphere (Figure 2(a)). The hearing subjects did not show such cross-modal activity, except for the lip-reading condition. Then, we conducted a two-way ANOVA to identify the difference in brain activity between the profoundly deaf, the residual hearing, and the hearing groups and between four visual conditions (Figure 2(b) and Table 3). Results demonstrated that the activations in the right STC had a significant main effect of both Group (*p* < 0.001, *p*_FDR_ < 0.05 corrected, Figure 2(b)) and Condition (*p* < 0.001, *p*_FDR_ < 0.05 corrected, Figure 2(b)). Other brain areas, including the bilateral middle lateral occipital gyrus, bilateral anterior temporal cortex (ATC), and inferior frontal gyrus (IFG), were also activated to the main effect of Condition (Table 3).

We next studied the STC activation in the right hemisphere that was induced by all four visual stimuli (Figures 2(a) and 2(c) and Table 2). For the checkerboard stimulus, we found that the right STC was significantly activated, and the post hoc region of interest (ROI, selected from the map showing the main effect of Group) analysis showed that the cross-modal activation was significantly higher in both the profoundly deaf (*t*-test, *p* < 0.017) and the residual hearing groups (*t*-test, *p* < 0.002) than in the hearing group (Figure 2(c), first row). For the visual word stimulus, the activation in the right STC showed significant differences between the profoundly deaf and the hearing groups (*t*-test, *p* < 0.002) and between the residual- and hearing groups (*t*-test, *p* < 0.001) (Figure 2(c), second row). For the sign language stimulus, the STC showed enhanced responses in both the profoundly deaf (*t*-test, *p* < 0.003) and the residual hearing groups (*t*-test, *p* < 0.02) in comparison with the hearing subjects (Figure 2(c), third row). For the lip-reading stimulus, cross-modal activations were found in the right STC in all subject groups, with no significant differences being found between the profoundly deaf and the hearing groups and between the residual hearing and the hearing groups (*t*-test, all *p* > 0.2; Figure 2(c), last row).

### 3.2. Correlations between Developmental Parameters and STC Activations

We then wished to investigate whether activations in auditory regions showed a stronger correlation with the duration of hearing loss or with the age of starting to learn sign language. Most of the residual hearing subjects had a reasonably similar learning duration in reading Chinese words and frequently used multiple language strategies (sign language, speech reading, and spoken language) in their communications. Thus, it is difficult to determine the accurate duration of language learning in the residual hearing group. In the correlation analysis, we only included profoundly deaf subjects and the developmental factors of duration of deafness and the age of learning sign language (Table 1). We first confirmed that the two developmental parameters were not significantly correlated with each other (Spearman's rank, *r* = −0.238, *p* > 0.392).

**Figure 3:**
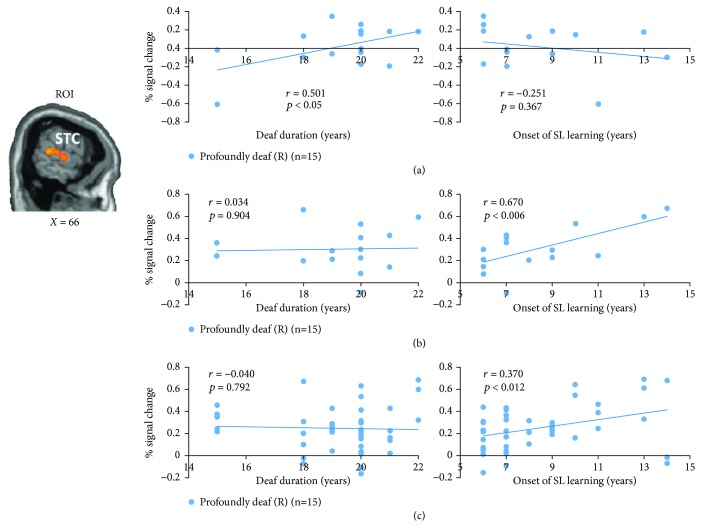
Correlations between superior temporal cortex (STC) activations and duration of deafness and onset of sign language learning in the profoundly deaf group. (a) A sagittal slice depicting the STC region of interest (ROI, selected based on the brain map showing the Group effect, Figure 2(b)) in the right hemisphere. Significant correlations were found in the profoundly deaf group (*n* = 15) between right STC activity for the checkerboard and duration of deafness (*r* = 0.501, *p* < 0.05, Bonferroni corrected), but not between this activation and the onset of sign language learning (the age of starting to learn sign language) (*r* = −0.251, *p* = 0.367). (b) In contrast, significant positive correlation was found between the activation for sign language and the onset of sign language learning (*r* = 0.670, *p* < 0.006, corrected), but not between the activation and hearing loss duration (*r* = 0.034, *p* = 0.904). (c) In the profoundly deaf group (*n* = 15), the STC activity induced by all the visual language stimuli (including words, sign language, and lip-reading) was correlated with the onset of sign language learning (*r* = 0.370, *p* < 0.012, corrected), but not with the duration of deafness (*r* = −0.04, *p* = 0.792).

For profoundly deaf individuals, we found that the right STC activation resulting from the checkerboard was positively correlated with the duration of deafness (Spearman's rank, *r* = 0.501, *p* < 0.05), but not with the age of sign language learning (Spearman's rank, *r* = −0.251, *p* > 0.366; Figure 3(a)). In contrast, the STC activation evoked by sign language stimuli was positively associated with the onset of sign language learning (*r* = 0.670, *p* < 0.006), but not with the duration of deafness (*r* = 0.034, *p* > 0.903; Figure 3(b)). Similar correlations were also found for all the visual stimuli that contained language content. That is, STC activity induced by all of the language stimuli was highly correlated with the onset of sign language learning (Spearman's rank, *r* = 0.370, *p* < 0.012), but not with the duration of deafness (Spearman's rank, *r* = −0.04, *p* > 0.792; Figure 3(c)). Further analyses showed that the activation in the left ATC and left IFG during the presentation of sign language was highly correlated with the onset of sign language learning (ATC: *r* = 0.642, *p* < 0.01; IFG: *r* = 0.703, *p* < 0.003; Figure S1). Interestingly, the activation in the same IFG region under the word condition also demonstrated a significant correlation with the onset of sign language learning (IFG: *r* = 0.501, *p* < 0.05) (Figure S1). However, no areas showing significant correlation with the onset of sign language were found under the checkerboard condition.

### 3.3. Whole-Brain Functional Connectivity with Cross-Modal Activity in the STC

**Figure 4:**
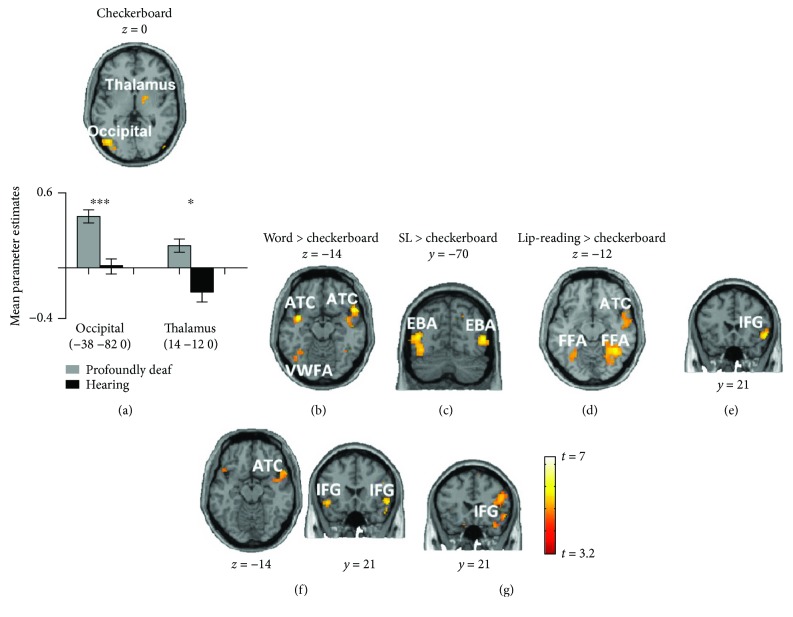
Differences in functional connectivity of the superior temporal cortex (STC) between the profoundly deaf and the hearing groups and between the checkerboard and the language conditions. Brain slices depicting the significant differences in functional connectivity between the profoundly deaf and the hearing groups (*p* < 0.001, FDR *p* < 0.05 corrected) with the seed region set in the right STC. For the checkerboard stimulus (a), significantly enhanced functional connectivity between profoundly deaf and hearing subjects was found in the left occipital cortex and right thalamus. In comparison with the checkerboard (whole brain, *p* < 0.001, FDR *p* < 0.05 corrected), for the word condition (b, e), greater connectivity was shown in the visual word form area, bilateral anterior temporal cortex, and right inferior frontal gyrus. For sign language (c, f), the bilateral extrastriate body area, right ATC, the bilateral FFA, and the bilateral IFG were identified as having strong functional connectivity with the right STC. For lip-reading stimuli (d, g), the bilateral FFA, right ATC and right IFG showed higher functional connectivity with the right STC. Asterisks denote a significant difference between the profoundly deaf group and the hearing group. Error bars indicate 1 standard error. ^∗∗∗^ *p* < 0.001 and ^∗^ *p* < 0.05.

We next examined the neural sources of cross-modal plasticity in the auditory cortex. We placed a seed region in the reorganized right STC and examined the difference in whole-brain functional connectivity between the profoundly deaf and the hearing subjects (*p* < 0.001, FDR *p* < 0.05 corrected) under the checkerboard condition. We identified significantly greater connection strengths to the STC in the occipital cortex (peak at $\left[\begin{array}{ccc} -38 & -82 & 0 \end{array}\right]$, *t*-test, *p* < 0.001) and right thalamus (peak at $\left[\begin{array}{ccc} 14 & -12 & 0 \end{array}\right]$, *t*-test, *p* < 0.01) of deaf subjects in comparison with hearing subjects (Figure 4(a)).

To explore the difference in functional connectivity between the language stimuli and the checkerboard, we further compared the connectivity contrast (profoundly deaf versus hearing) of each language stimulus with the checkerboard contrast at the whole-brain level (*p* < 0.001, FDR *p* < 0.05 corrected) (Figure 4 and Table 4). For the word stimuli, compared with the checkerboard, we found enhanced connection strengths not only in the left occipital cortex (left hemisphere, peak at $\left[\begin{array}{ccc} -42 & -58 & -14 \end{array}\right]$, *t* = 3.89) but also in the bilateral ATC (the left hemisphere: peak at $\left[\begin{array}{ccc} -40 & 2 & -14 \end{array}\right]$, *t* = 5.67; the right hemisphere: peak at $\left[\begin{array}{ccc} 58 & 14 & -14 \end{array}\right]$, *t* = 4.97) and right IFG (peak at $\left[\begin{array}{ccc} 54 & -21 & 3 \end{array}\right]$, *t* = 8.73; Figures 4(b) and 4(e)). The connected area in the left occipital cortex for the word condition was located precisely in the classical visual word form area, which is specific to the processing of visual word information [43–45]. For the sign language stimuli, we identified significantly stronger connections in the bilateral middle temporal areas (the right hemisphere: peak at $\left[\begin{array}{ccc} 52 & -68 & 6 \end{array}\right]$, *t* = 6.84; the left hemisphere: peak at $\left[\begin{array}{ccc} 44 & -70 & 8 \end{array}\right]$, *t* = 5.74), the bilateral FFA (the right hemisphere: peak at $\left[\begin{array}{ccc} 42 & -52 & -16 \end{array}\right]$, *t* = 5.70; the left hemisphere: peak at $\left[\begin{array}{ccc} -32 & -60 & -16 \end{array}\right]$, *t* = 4.20), right ATC (peak at $\left[\begin{array}{ccc} 50 & 12 & -14 \end{array}\right]$, *t* = 5.63), and bilateral IFG (the right hemisphere: peak at $\left[\begin{array}{ccc} 54 & 21 & -6 \end{array}\right]$, *t* = 5.83; the left hemisphere: peak at $\left[\begin{array}{ccc} -48 & 21 & -10 \end{array}\right]$, *t* = 5.33; Figures 4(c) and 4(f)). The activated bilateral visual areas were identified to be selective for visual processing of the human body (extrastriate body area, EBA) [46]. For the lip-reading condition, we found significantly greater connection strengths in the bilateral FFA (the right hemisphere: peak at $\left[\begin{array}{ccc} 38 & -58 & -12 \end{array}\right]$, *t* = 7.38; the left hemisphere: peak at $\left[\begin{array}{ccc} -26 & -56 & -10 \end{array}\right]$, *t* = 5.88, Figure 4(d)), right ATC (peak at $\left[\begin{array}{ccc} 58 & -2 & -10 \end{array}\right]$, *t* = 6.13), and right IFG (peak at $\left[\begin{array}{ccc} 52 & 21 & 20 \end{array}\right]$, *t* = 3.80; Figure 4(g)). The FFA, which is well known as an area involved in the processing of face information [47], was activated in both the sign language and the lip-reading conditions. In short, in comparison with the checkerboard stimulus, the STC activity induced by language stimuli received extra and common connections from the ATC (e.g., the temporal pole) and frontal (e.g., IFG) regions. Additionally, the sensory component was mainly from visual areas (including the VWFA, EBA, and FFA) that seemed highly selective to stimulus features.

### 3.4. Dynamic Causal Modeling

**Figure 5:**
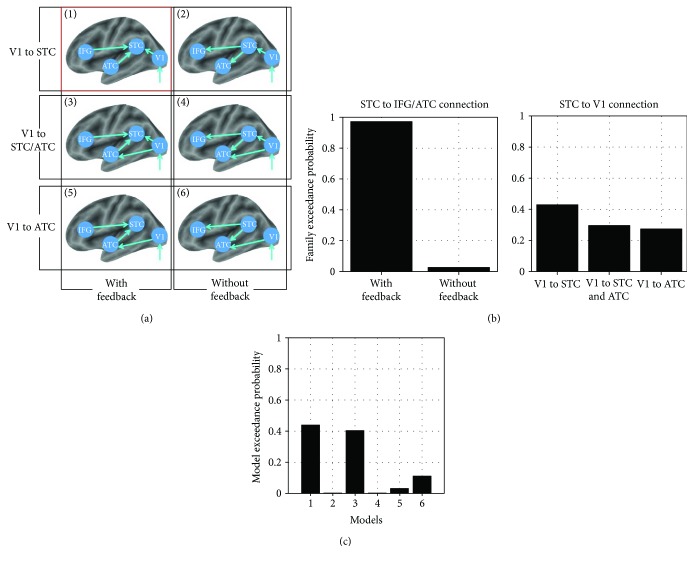
Dynamic causal modeling. (a) The six dynamic causal models used for Bayesian model comparison. Each model receives (parameterized) input at primary visual cortex (V1) source under the language condition in deaf participants. (b) Family-wise Bayesian model selection was used to establish the best neural network architecture for the feedback and feed-forward effect to the STC. Families of models with feedback from IFG/ATC to STC and with feed-forward from V1 to STC/ATC best explained the data. (c) Random effects Bayesian model selection showed model 1 (marked with the red box in (a)) best fits the data for the language condition in deaf individuals.

Although we found that the visual cortical areas, ATC, and IFG showed functional connections with STC under the language condition, we still do not know the causal direction between these brain regions. Dynamic causal modeling (DCM) is a generic Bayesian framework for inferring interactions among hidden neural states from measurements of brain activity and has been used in early blind individuals [48, 49]. Thus, we used DCM and Bayesian model selection to explore how language components reach the STC in deaf subjects by comparing six plausible models (Figure 5(a)). Random effects Bayesian model selection showed that cross-modal activity observed in the STC of deaf subjects was best explained by the feedback connection from IFG/ATC (Figure 5(b), left, with feedback; exceedance probability of 0.97) and feed-forward connection from V1 (Figure 5(b) right, V1 to STC; exceedance probability of 0.43; and V1 to STC/ACT; exceedance probability of 0.30) (in model 1, Figure 5(c); exceedance probability of 0.44). The result strongly suggested that the feedback component from language circuit (ATC and IFG) and the feed-forward component from the sensory region were both involved in the induction of cross-modal plasticity in the STC under the language condition.

### 3.5. Representational Similarity of Cross-Modal Activation in the STC

**Figure 6:**
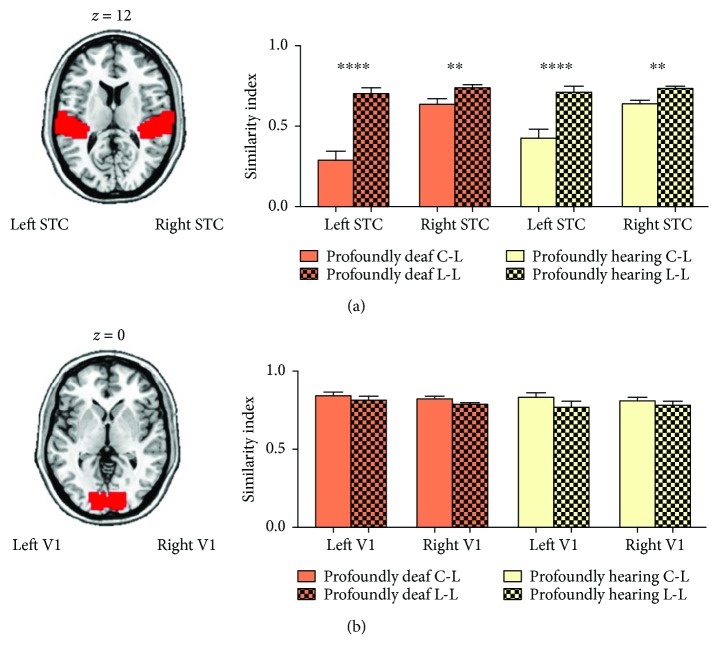
Representational similarities in activation patterns in the superior temporal cortex (STC) and primary visual cortex. (a) Axial slice depicting the auditory region of interest (ROI; Brodmann areas (BAs) 41 and 42) in red. The bars indicate the similarity (correlation coefficient index) of the spatial activation patterns between visual stimuli within the ROI. The solid bars depict the averaged similarities between checkerboard and language-related stimuli (C-L), including sign language, words, and lip-reading, in both the profoundly deaf group (in orange) and the residual hearing group (in yellow). The grid bars depict the average correlation coefficients between language-related stimuli (L-L). The correlation coefficient between language-related stimuli in the bilateral auditory ROIs was significantly higher than that between checkerboard and language-related stimuli in both the profoundly deaf group (left hemisphere: *p* < 0.0001, right hemisphere: *p* < 0.001) and the residual hearing group (left hemisphere: *p* < 0.0001, right hemisphere: *p* < 0.0016). (b) Such differences in representational similarity were not observed in the primary visual cortex (V1; BA17). ^∗∗^ *p* < 0.01; ^∗∗∗∗^ *p* < 0.0001. Error bars represent 1 standard error.

We finally wanted to explore whether cross-modal activities in the STC shared the same spatial activity pattern when in receipt of distinct contributions from occipital and temporal-frontal areas. We used a multivariate pattern analysis technique known as representational similarity analysis (RSA) [39] to examine how the spatial pattern of BOLD signals over voxels varied in response to different visual stimuli. Per subject, the representational dissimilarity matrixes (RDMs) comprised correlation distances (1 correlation coefficient) between the images from the blocks for each condition in both the profoundly deaf and the residual hearing groups, which yielded a 4 × 4 matrix. The four conditions were separated into two categories: nonlanguage (checkerboard) and language (words, sign language, and lip-reading). We then compared the correlation coefficient in the three pairs between the nonlanguage and the language conditions (category C-L) with the three pairs within the language conditions (category L-L) for each subject. Results showed that correlation coefficients between the checkerboard and any of the language-related stimuli in the bilateral STC were significantly lower than those between any two language-related stimuli in both the profoundly deaf (Figure 6(a), the left hemisphere, *t*-test, *p* < 0.0001; the right hemisphere, *t*-test, *p* < 0.005) and the residual hearing groups (Figure 6(a), the left hemisphere, *p* < 0.0001; the right hemisphere, *p* < 0.05). As a control comparison, no significant differences in RSA were found in the primary visual cortex in either the profoundly deaf (Figure 6(b), the left hemisphere, *t*-test, *p* > 0.65; the right hemisphere, *t*-test, *p* > 0.81) or the residual hearing individuals (Figure 6(b), the left hemisphere, *t*-test, *p* > 0.72; the right hemisphere, *t*-test, *p* > 0.87).

## 4. Discussion

Relative to hearing subjects, both profoundly deaf and residual hearing subjects showed enhanced STC responses to checkerboard, word, and sign language stimuli, which confirmed the existence of cross-modal plasticity after auditory deprivation [2, 14, 35, 50, 51]. While Lambertz et al. [51] reported that cortical reorganization of the auditory cortex was only present in profoundly deaf subjects not in subjects with residual hearing ability, our results showed that such plasticity existed in both groups of hearing-impaired subjects. One possible interpretation could be that intensive behavioral and perceptual training caused neuroplasticity in the late-onset sensory deprivation [30]. Despite the fact that there are differences between pre- and postlingually deaf individuals, cross-modal activity is consistently found in postlingually deaf CI patients as well as in mild to moderately hearing impaired individuals [33, 52–54]. Hearing subjects also showed significant STC responses to lip-reading in the present study, which is compatible with previous observations indicating that silent speech reading activates lateral parts of the superior temporal plane in hearing adults [15, 20–23].

Although sensory deprivation triggers cortical reorganization, the origin of anatomical and functional changes observed in the STC of deaf individuals is not only sensory (feed-forward) but also cognitive (feedback), such as in the use of sign language and speech reading [30]. The purely visual stimulus (checkerboard) provoked activations in the right STC, which showed correlations only with the duration of deafness [52], and strong functional connectivity with the visual cortex and thalamus, implying the contribution of sensory components to the plasticity. However, the cognitive stimuli with language content induced activations in both the right and the left STC, which exhibited strong association only with the experience of sign language learning, and enhanced functional connections with not only visual cortical areas but also the ATC and IFG, suggesting a strong potential top-down modulation of plasticity induced by the linguistic components of the cognitive stimuli. The DCM analysis further confirmed the information flow triggered by the visual stimuli with language content, by showing a strong feedback effect from IFG/ATC and a feed-forward effect from V1 to STC.

In deaf humans, it was found that auditory areas preserved the task-specific activation pattern independent of input modality (visual or auditory), suggesting the task-specific reorganization during the cortical plasticity [55]. Cardin et al. [18] showed that auditory deprivation and language experience cause activations of different areas of the auditory cortex when two groups of deaf subjects with different language experience are watching sign language. In the auditory cortical areas of deaf animals, Lomber with his colleagues showed that the neural basis for enhanced visual functions was located to specific auditory cortical subregions. The improved localization of visual stimuli was eliminated by deactivating the posterior auditory cortex, while the enhanced sensitivity to visual motion was blocked by disabling the dorsal auditory cortex [25, 56, 57]. Land et al. [58] demonstrated that visually responsive and auditory-responsive neurons in the higher-order auditory cortex of deaf cats form two distinct populations that do not show bimodal interactions. However, we still know little of how other brain regions contribute to the task-specific activations in the auditory cortex. In the present study, although neural reorganization in deaf individuals permits both the sensory and language inputs to reach the STC, the RSA result suggested that the functions of the two components were segregated within the reorganized auditory area, which confirmed the functional segregation hypothesis. Furthermore, our functional connectivity analysis suggested that the stimulus-specific activation in STC was probably developed via different neural pathways. Specifically, the sensory component of stimuli was found to be highly stimulus-specific. During the word presentation, visual areas functionally connected with the STC were located exactly within the visual word form area (only in the left hemisphere, Figure 4(b)) which is a region demonstrated to be involved in the identification of words and letters from lower-level shape images prior to association with phonology or semantics [44, 45, 59]. During the sign language and lip-reading stimuli, the functionally connected visual areas were identified as being in the extrastriate body area and fusiform face area, which is known to be especially involved in facial recognition [47, 60] and human body representation [46]. In contrast, for the language component, the cross-modal plasticity shaped by sign language could also be generalized to the responses to other language stimuli (Figure 3(c) and Figure S1). Additionally, STC activities induced by the word, sign language, and lip-reading stimuli were functionally connected with a similar neural network consisting of the temporal pole areas and inferior frontal regions (part of Broca's area) (Figure 4), which was shown to be involved in semantic processing in the language system [61]. These results may suggest that the language components from different visual stimuli share a common language circuit for top-down visual-auditory reorganization.

The difference between checkerboard and language-related stimuli (Figure 6(a)) cannot be interpreted by other experimental accounts. For example, one may argue that the language stimuli have a higher visual richness than the purely visual stimuli, which may therefore have induced higher similarity within language stimuli. This seems unlikely, as such a difference in similarity was not shown in the primary visual cortex (Figure 6(b)).

Previous studies on prelingual deaf groups have proposed a link between poor speech outcomes and exposure to a visual language and indicate that the incapacity for processing auditory signals (poorer outcomes of cochlear implant) is due to usurping of the auditory cortex functionality by visual language [31, 32, 62–64]. However, to the contrary, some studies indicate that proficiency with speech reading is linked to better outcomes of cochlear implant [31, 33, 65–68]. Thus, together with other animal studies, our human imaging results on functional segregation suggest that, although exposure to sign language may indeed partially take over the auditory cortex, the auditory regions could still preserve the ability to process auditory signals following cochlear implants, with this being facilitated through the recruitment of new populations of neurons or different spatial activity patterns.

In conclusion, both language and sensory components contribute to the cross-modal plasticity of the STC in deaf people; these are associated with hearing loss duration and language experience, respectively. The feed-forward signal of sensory input is highly stimulus-specific, while the feedback signal from language input is more associated with a common neural network. Finally, even though both pathways activate auditory areas in deaf people, they seem functionally segregated in respect to cross-modal plasticity. In summary, this study provides important and unique evidence for understanding the neural circuits involved in cross-modal plasticity in deaf people and may guide clinicians in consideration of cochlear implants or hearing recovery.

## Figures and Tables

**Table:** (a) Profoundly deaf

Number	Age (years)	Sex	Cause of deafness	Hearing threshold (dB)		Age of SL (years)	Duration of deafness (years)
				Left	Right		
1	17	M	Hereditary	104	106	N/A	16
2	20	M	Ototoxic drugs	110	110	6	19
3	20	M	Ototoxic drugs	84	79	8	18
4	24	M	Ototoxic drugs	119	118	13	22
5	21	F	Ototoxic drugs	112	108	10	20
6	22	F	Ototoxic drugs	118	120	11	15
7	18	F	Ototoxic drugs	119	120	7	15
8	20	M	Ototoxic drugs	110	93	9	19
9	19	F	Hereditary	109	109	N/A	18
10	20	M	Hereditary	94	93	N/A	20
11	23	F	Meningitis	103	108	6	21
12	22	F	Hereditary	81	95	N/A	22
13	21	F	Unknown	110	109	7	19
14	22	F	Ototoxic drugs	108	117	7	21
15	21	M	Ototoxic drugs	110	110	6	21
16	21	F	Meningitis	104	103	6	20
17	22	M	Ototoxic drugs	105	110	7	20
18	22	M	Ototoxic drugs	>120	109	6	21
19	21	M	Ototoxic drugs	>110	110	13	19

**Table:** (b) Residual hearing

Number	Age (years)	Sex	Cause of deafness	Hearing threshold (dB)		Duration of deafness (years)	Duration of hearing aid use (years)
				Left (without aid)	Right (without aid)		
1	18	F	Meningitis	60 (105)	55 (101)	18	15
2	19	M	Ototoxic drugs	100	75 (75)	17	14
3	19	F	Ototoxic drugs	48 (73)	35 (70)	19	10
4	20	F	Ototoxic drugs	105	79 (106)	18	10
5	19	M	Meningitis	41 (88)	64 (103)	19	15
6	24	M	Ototoxic drugs	70 (76)	93 (101)	23	11
7	24	F	Head injury	66 (107)	94 (98)	22	22
8	24	F	Ototoxic drugs	47 (68)	65 (93)	23	20
9	21	F	Ototoxic drugs	75 (86)	89	19	14
10	20	M	Ototoxic drugs	69 (99)	75 (103)	18	17
11	21	F	Ototoxic drugs	86 (108)	66 (88)	19	18
12	19	F	Ototoxic drugs	69 (88)	84 (99)	18	15
13	20	F	Ototoxic drugs	96	83 (95)	18	14
14	20	F	Ototoxic drugs	108	84 (110)	18	11
15	23	F	Ototoxic drugs	65 (101)	101	20	19

**Table:** (c) Hearing participants

Number	Age (years)	Sex
1	19	M
2	23	M
3	19	F
4	17	F
5	19	F
6	21	F
7	20	F
8	20	F
9	19	M
10	19	F
11	19	F
12	24	M
13	22	M
14	22	M
15	22	M

The hearing loss of deaf participants was confirmed by testing hearing thresholds with audiometry (see Methods and Materials) in the Institute of Speech and Hearing Science at East China Normal University. The averaged hearing thresholds in decibels of each participant are reported in the table. Profoundly deaf group: *n* = 19, 9 females, mean age = 20.84 ± 1.68 years; residual hearing group: *n* = 15, 11 females, mean age = 20.73 ± 2.05 years. SL: sign language. Note: ototoxic drugs mean the misuse of antibiotics.

**Table 2:** Peak activations for BA41 and BA42: profoundly deaf, residual hearing, and hearing groups (*p* < 0.001, uncorrected, minimum cluster size = 10).

Group	Peak coordinates (BA41)			Number of voxels	Peak *Z* statistic	Peak coordinates (BA42)			Number of voxels	Peak *Z* statistic
	*X*	*Y*	*Z*			*X*	*Y*	*Z*		
Checkerboard										
Profound	/	/	/	/	/	66	−27	15	19	4.47
Residual	/	/	/	/	/	69	−24	9	30	3.87
Hearing	/	/	/	/	/	/	/	/	/	
Word										
Profound	−48	−33	12	23	4.49	60	−30	15	29	3.78
Residual	/	/	/	/	/	69	−27	9	12	3.64
Hearing	/	/	/	/	/	/	/	/	/	
Sign language										
Profound	54	−24	6	20	4.54	66	−30	12	34	5.54
	/	/	/	/	/	−66	−30	6	11	4.75
Residual	48	−33	9	24	5.07	66	−36	18	31	4.62
	/	/	/	/	/	−63	−30	6	18	4.75
Hearing	/	/	/	/	/	/	/	/	/	
Lip-reading										
Profound	54	−24	6	16	4.72	66	−24	12	39	4.70
	/	/	/	/	/	−66	−30	6	13	3.92
Residual	57	−27	12	18	4.41	63	−24	12	45	5.10
	/	/	/	/	/	−69	−27	6	38	5.36
Hearing	54	−24	6	13	4.44	66	−30	12	33	4.40
	/	/	/	/	/	−69	−27	6	14	4.26

Peak coordinates refer to stereotactic coordinates in MNI (Montreal Neurological Institute) space. BA: Brodmann area.

**Table 3:** Peak activations for the main effect of Group and Condition (two-way ANOVA, *p* < 0.001, FDR *p* < 0.05 corrected).

Main effect	Brain region	Number of voxels	Peak coordinates			*Z* score
			*X*	*Y*	*Z*	
Group	Temporal_Superior_Right	151	66	−27	13	4.16
	Occipital_Middle_Left	53	−36	−81	0	4.14
	Occipital_Middle_Right	77	24	−96	3	5.49
	Anglar_Left	34	−54	−60	39	4.02
	Frontal_Middle_Right	21	−33	6	45	4.01
	Precentral_Left	56	−24	−3	36	3.76
	Thalamus_Right	10	24	−27	21	3.92
	Cerebelum_7b_Left	17	−36	−48	−42	3.47
	SupraMarginal_Right	19	42	−39	33	3.35
	Cingulum_Anterior_Right	10	6	42	15	3.17

Condition	Temporal_Superior_Right	426	66	−25	10	6.75
	Temporal_Superior_Left	305	−63	−48	9	7.54
	Frontal_Inferior_Tri_Left	249	−48	22	13	7.58
	Frontal_Inferior_Tri_Right	45	42	30	0	5.85
	Temporal_Middle_Left	234	54	−3	45	6.37
	Temporal_Middle_Right	261	51	−66	3	5.75
	Temporal_Pole_Left	34	−57	7	−9	4.47
	Temporal_Pole_Right	50	57	6	−12	5.19
	Cingulum_Anterior	123	−12	42	0	7.36
	ParaHippocampal_Right	56	30	−36	−9	7.05
	Fusiform_Left	30	−30	−39	−12	6.44
	Frontal_Middle_Right	23	30	24	39	4.12
	Precentral_Left	54	−51	−6	48	5.41
	Precentral_Right	12	54	−3	45	5.19
	Precuneus_Right	49	9	−48	48	6.22
	Lingual_Left	29	0	−78	−3	4.68

Note: peak coordinates refer to stereotactic coordinates in MNI space.

**Table 4:** Difference in functional connections with the right STC as a seed region.

Contrast	Brain region	Number of voxels	Peak coordinates			*T* value
			*X*	*Y*	*Z*	
Checkerboard: profoundly deaf versus hearing group	Occipital_Middle_Left	399	−38	−82	0	5.23
	Occipital_Middle_Left	318	−24	−94	8	5.21
	Cerebellum_Left	40	−32	−60	−48	4.71
	ParaHippocampal_Right	15	30	−16	−28	4.38
	Cingulum_Middle_Right	58	10	26	32	4.15
	Thalamus_Right	35	14	−12	0	3.89
	Supplementary_Motor_Right	11	12	18	60	3.73
	Cerebellum_Left	28	−30	−58	−24	3.71

Word: (word: profoundly deaf versus hearing groups) versus (checkerboard: profoundly deaf versus hearing)	Frontal_Inferior_Orb_Right	74	54	21	3	6.67
	Temporal_Pole_Superior_Right	54	58	14	−16	6.02
	Temporal_Pole_Superior_Left	49	−40	2	−16	4.95
	Visual_word_form_area_Left	47	−42	−58	−14	4.71
	Occipital_Middle_Right	32	42	−74	14	4.06
	Cingulum_Middle_Right	23	6	4	40	3.88

Sign language: (sign language: profoundly deaf versus hearing groups) versus (checkerboard: profoundly deaf versus hearing)	Occipital_Middle_Left	253	−44	−70	8	5.98
	Occipital_Middle_Right	124	52	−68	6	6.84
	Fusiform_Left	67	−32	−60	−16	4.20
	Fusiform_Right	51	42	−52	−16	5.70
	Temporal_Pole_Superior_Right	44	50	12	−14	5.89
	Temporal_Pole_Superior_Left	19	−44	14	−16	4.13
	Frontal_Inferior_Orb_Left	37	54	21	−6	4.31
	Frontal_Inferior_Orb_Right	30	−48	21	−10	5.83
	Supplementary_Motor_Area	39	6	10	64	4.39
	Parietal_Superior_Left	12	−20	−54	50	4.23

Lip-reading: (lip-reading: profoundly deaf versus hearing groups) versus (checkerboard: profoundly deaf versus hearing)	Fusiform_Left	122	−26	−56	−10	5.45
	Fusiform_Right	146	38	−58	−12	7.85
	Temporal_Pole_Superior_Right	97	58	−2	−10	5.77
	Frontal_Inferior_Tri_Right	43	48	22	18	3.79
	Frontal_Inferior_Orb_Right	21	52	21	20	3.53
	Precentral_Left	19	−44	−6	−48	4.25
	Precentral_Right	22	52	4	46	6.87
	Supplementary_Motor_Area	13	−4	6	60	5.13

Note: peak coordinates refer to stereotactic coordinates in MNI space; *p* < 0.001, FDR *p* < 0.05 corrected.

## Supplementary Materials

Supplementary Materials Supplemental information includes one figure, Supplemental visual materials, and Supplemental questionnaire (translated to English) after scanning.
