# Supplementary material for: Language and Sensory Neural Plasticity in the Superior Temporal Cortex of the Deaf
Source: Neural Plast. 2018 May 2;2018:9456891. doi: 10.1155/2018/9456891 (PMC5954881; doi:10.1155/2018/9456891)
Supplement: Supplementary Materials — Supplemental information includes one figure, Supplemental visual materials, and Supplemental questionnaire (translated to English) after scanning. [file 9456891.f1.pdf]

Supporting Figure 1

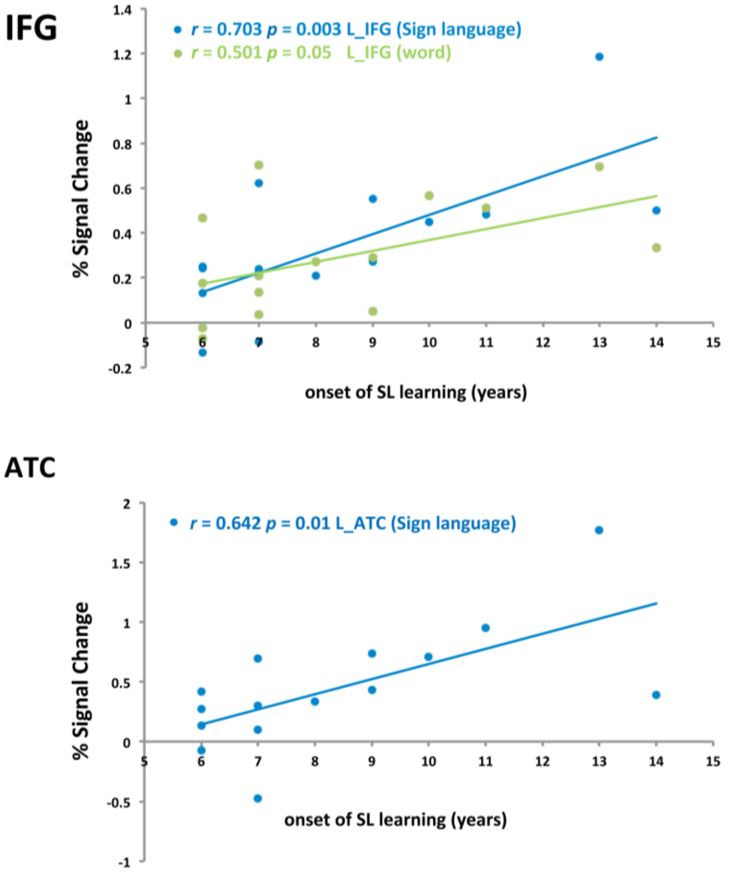

Figure S1. Correlations between inferior frontal gyrus (IFG) and anterior temporal cortex (ATC) activations and onset of sign language learning in the profoundly deaf group. Significant positive correlations were found in the profoundly deaf group (n = 15) between left IFG activity for both the sign language (r = 0.703, p < 0.003) and word (r = 0.501, p < 0.05) and onset of sign language learning. Significant correlation was also found between left ATC activity for sign language and onset of sign language learning (r = 0.642, p < 0.01).

Visual stimuli

Checkerboard

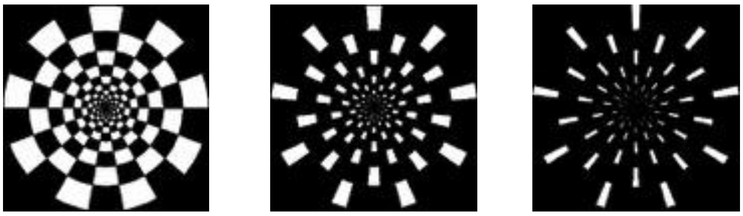

A

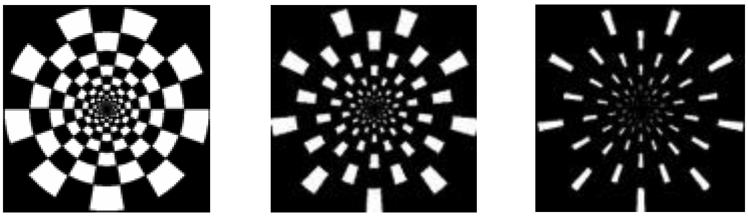

B

The checkerboard consisted of reversing displays of checkerboard patterns, based on a monochrome image pair (as shown above). The image pair of each stimulus is referred to as Images A and B. Image B was generated by rotating Image A through 180°. All stimuli (1280 × 1024 pixels) had a radial nature and consisted of 20 rings. Each image was presented for one second. Images A and B changed at a reversal rate of 2 Hz. The contrast between white and black pixels was identical in all images used.

Words

我、的、被、千、是、花、等、所、你、这
上、白、想、妈、天、文、句、家、手、心
小、新、前、长、先、中、能、看、种、吃
问、站、孩、打、思、四、八、谁、读、学
周、要、住、描、您、现、较、跑、望、面
钱、每、到、令、画、物、山、同、别、岁
李、爱、指、国、各、请、船、照、门、对
那、正、里、帮、件、百、海、笔、随、书

Word (translated to English)

me, of, by, thousand, is, flower, wait, that, you, this
up, white, miss, mother, sky, language, home, hand, heart
small, new, front, long, before, middle, can, look, seed, eat
ask, stand, child, hit, think, four, eight, who, read, learn
week, want, live, trace, you, now, compare, run, visit, face
money, every, arrive, force, draw, thing, mountain, same, depart, year
plum, love, finger, country, each, please, boat, photo, door, right
there, positive, in, help, piece, hundred, sea, pen, follow, book

Sign language

你想买什么？我想看看那件衣服。
我能帮你吗？我想要一张上海地图。
生日快乐！新年好！圣诞快乐！祝你身体健康！
你们的房子真漂亮！
我能在这里抽烟吗？对不起，不能。

Sign language (translated to English)

What would you like to buy? I want to have a look at that dress.
Can I help you? I would like to have a map of Shanghai.
Happy Birthday! Happy New Year! Merry Christmas!
Your house is really beautiful!
Can I smoke here? I'm sorry, you can't.

Lip-reading

我们、他们、没有、问题、工作、这个、生活、时间、开始、开心
人民、成为、北京、历史、方向、结果、而且、学生、政府、果实
单位、群众、应当、个人、全国、意义、条件、环境、了解、力量
注意、当然、家庭、原因、进入、介绍、下来、事情、方式、计划
约定、飞行、应该、政治、公司、任务、出现、一切、研究、非常

Lip-reading (translated to English)

we, they, none, question, work, this, life, time, start, happy
people, become, Beijing, history, direction, result, furthermore, student, government, fruit
unit, public, must, individual, nationwide, meaning, condition, environment, understand, power
attention, of course, family, reason, access, introduce, come down, things, methods, plan
convention, fly, ought to, politics, company, task, emerge, everything, research, very much

Questionnaire (Translated to English)

Please tick your choices in the corresponding options from the following question

1. How many types of visual stimuli you have seen in the experiment?

3 # 4 # 5

2. Did you find the checkerboard stimuli clearly?

Clear # Not sure # Not clear at all

3. Did you find the words comprehensible?

Yes # Not sure # Incomprehensible

Please recall what words had been presented? e.g.

4. Did you find the sign language stimuli comprehensible?

Yes # Not sure # Incomprehensible

Please recall the content of sign language that had been presented? e.g.

5. Did you find the lip-reading stimuli comprehensible?

Yes # Not sure # Incomprehensible

Please recall the content of lip-reading that had been presented? e.g.

Please descript the content of your thoughts when there is no visual presentation. We also appreciate any suggestions about the experiment.

We appreciate your participation!
